# Stent-Free Excimer Laser Atherectomy in AMI With Aplastic Anemia and Thrombocytopenia

**DOI:** 10.1016/j.jaccas.2025.104658

**Published:** 2025-08-06

**Authors:** Yuehua Ma, Qinping Zhang, Wei Wang, Lei Gao

**Affiliations:** a Department of Cardiovascular Medicine, Xinxiang Central Hospital, Xinxiang, Henan, China; b Department of General Practice, Chinese People's Liberation Army Unit 66284 Hospital, Beijing, China; c Senior Department of Cardiology, The Sixth Medical Center of Chinese People's Liberation Army (PLA) General Hospital, Beijing, China

**Keywords:** acute myocardial infarction, aplastic anemia, excimer laser coronary atherectomy, thrombocytopenia

## Abstract

**Background:**

Acute myocardial infarction (AMI) complicated by aplastic anemia with severe thrombocytopenia presents a therapeutic dilemma because of conflicting bleeding and thrombotic risks.

**Case Summary:**

A 60-year-old man with aplastic anemia (platelet count: 28-32 × 10^9^/L) and ST-segment elevation myocardial infarction underwent excimer laser coronary atherectomy (ELCA) combined with a stent-free strategy. Coronary angiography revealed multivessel disease with total right coronary artery occlusion. ELCA reduced thrombus burden, restored TIMI flow grade 3, and avoided the need for dual antiplatelet therapy. Follow-up confirmed sustained patency and no bleeding complications.

**Discussion:**

This case highlights the efficacy of ELCA in resolving thrombotic burden while circumventing dual antiplatelet therapy and stent-related risks, addressing a literature gap in managing AMI with severe thrombocytopenia.

**Take-Home Messages:**

ELCA with a stent-free approach safely restores coronary flow in patients with thrombocytopenic AMI, balancing thrombus resolution and bleeding risks. This strategy offers a paradigm for high-risk cases where conventional therapies are contraindicated.

## Graphical Abstract

**Figure:**
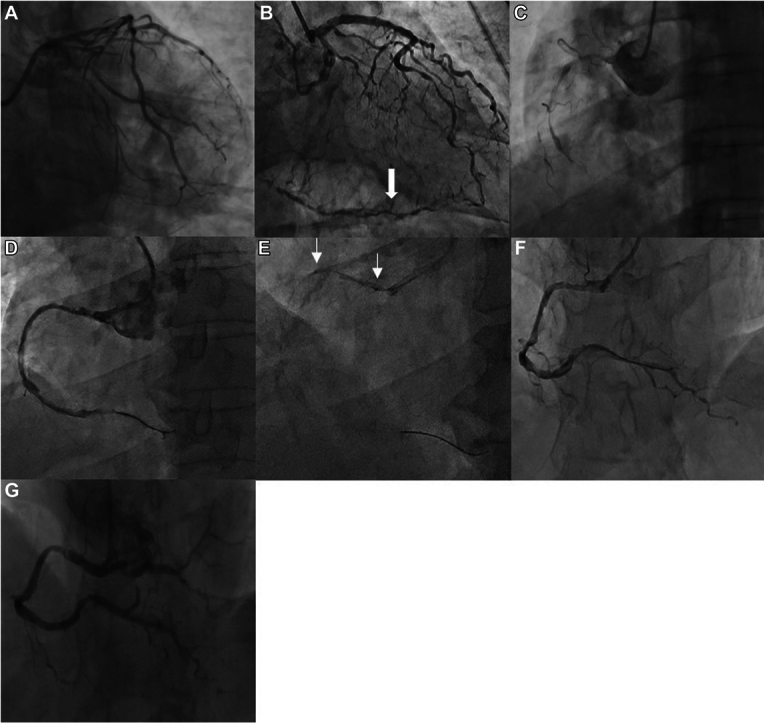

## History of Presentation

A 60-year-old man with a 20-year history of thrombocytopenia associated with aplastic anemia (AA; baseline platelet count: 30 × 10^9^/L) was referred to our institution for evaluation and treatment of recurrent chest pain persisting over 12 days. The patient had experienced 2 previous episodes of acute ST-segment elevation myocardial infarction (STEMI). However, he was deemed ineligible for percutaneous coronary intervention (PCI) or thrombolytic therapy at the referring center owing to severe thrombocytopenia. Upon admission, a hematology consultation recommended initiating clopidogrel monotherapy (75 mg/d) while continuing his prescribed hematologic regimen. A repeat laboratory evaluation showed platelet counts fluctuating between 28 and 32 × 10^9^/L.

**Take-Home Messages**

• ELCA with a stent-free approach safely restores coronary flow in patients with thrombocytopenic AMI, resolving thrombotic burden while minimizing bleeding risks.
• This strategy provides a viable alternative for high-risk cases where conventional PCI or DAPT is contraindicated, highlighting the value of tailored procedural and antithrombotic protocols.

## Past Medical History

The patient had a 20-year history of AA. His hematologic management included *Shengban zhixue* pills (*Panax notoginseng*, *Euphorbia humifusa*), which contain saponins/flavonoids, promoting platelet aggregation and anti-inflammatory hemostasis while resolving blood stasis (3 bags/d); *Shengbai* capsules (*Astragalus membranaceus*, *Codonopsis pilosula*), which use polysaccharides/saponins to stimulate hematopoiesis and immunity, targeting leukopenia and *qi*-blood deficiency (9 capsules/d); *Qihuang zhixue* capsules (*Astragalus*, donkey-hide gelatin, *Epimedium*), which enhance leukocyte/hemoglobin production via blood-*qi* tonification (9 capsules/d); and *Chailu shugan jiedu* capsules (*Bupleurum*, *Artemisia capillaris*) to alleviate liver *qi* stagnation through choleretic and anti-inflammatory mechanisms (9 capsules/d); this regimen maintained his platelet count above 25 × 10^9^/L without any signs of bleeding. Additional comorbidities included primary hypertension, type 2 diabetes mellitus, and a cerebral infarction 5 years earlier. Notably, long-term antiplatelet therapy had been avoided owing to the elevated risk of bleeding associated with his thrombocytopenia.

## Differential Diagnosis

The patient presented with chest pain, and electrocardiogram revealed inferior wall ST-segment elevation accompanied by elevated cardiac biomarkers (including troponin), confirming the diagnosis of acute STEMI.

## Investigations

An initial bolus of 20 mg unfractionated heparin was administered at the initiation of coronary angiography, with activated clotting time monitored to guide subsequent dosing adjustments. During the PCI procedure, an additional 40 mg was titrated to maintain the activated clotting time within the target range of 250 to 300 seconds.

**Figure 1:**
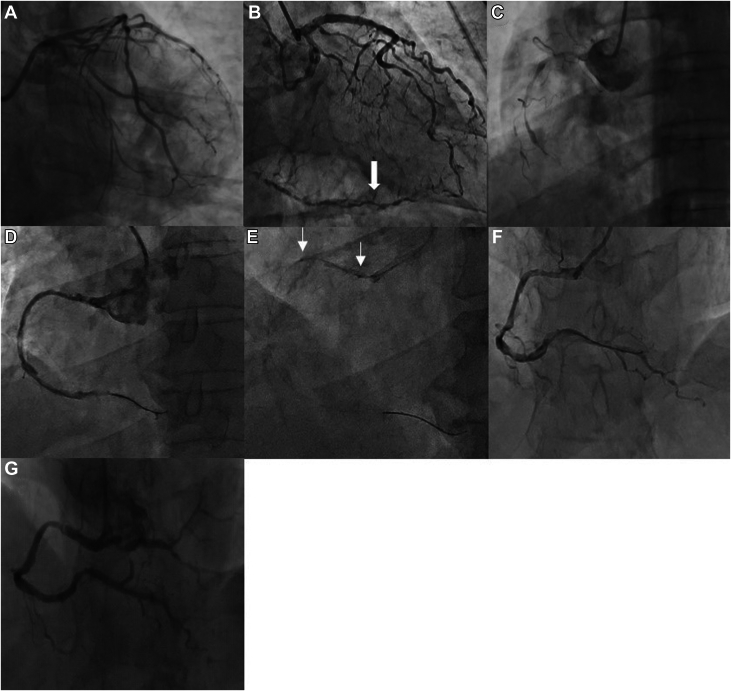
Coronary Angiographic and Procedural Images (A and B) Coronary angiography showed 70% diffuse stenosis in the distal left anterior descending artery and left circumflex branch; the arrow indicates collateral vessels between the left coronary artery and right coronary artery. (C) Severe stenosis in the right coronary artery with complete distal occlusion in the proximal right coronary artery. (D) After excimer laser coronary angioplasty (ELCA). (E) Balloon angioplasty site (post-ELCA) between markers (arrows). (F) After balloon dilation, angiography showed that the stenosis was significantly reduced and the forward flow was TIMI flow grade 3. (G) Coronary angiography at 15 days postprocedure.

Coronary angiography revealed multivessel disease, including 70% diffuse stenosis of the distal left anterior descending artery and left circumflex artery, as well as 90% diffuse stenosis with complete distal occlusion in the proximal right coronary artery (Figures 1A to 1C). Because of the high risk of bleeding, intracoronary thrombolysis and glycoprotein IIb/IIIa inhibitors were contraindicated. Intervention on the right coronary artery was initiated with multiple manual thrombus aspirations, which were implemented as follows: After successful guidewire traversal across the lesion, the aspiration catheter was advanced over the guidewire to a position distal to the thrombotic occlusion. After securing the negative pressure system, the catheter was gradually retracted proximally while continuous manual aspiration was applied through repeated syringe activation to ensure optimal thrombus removal. Despite these efforts, only minimal thrombus was extracted, and TIMI flow grade 0 persisted.

## Management

**Figure 2:**
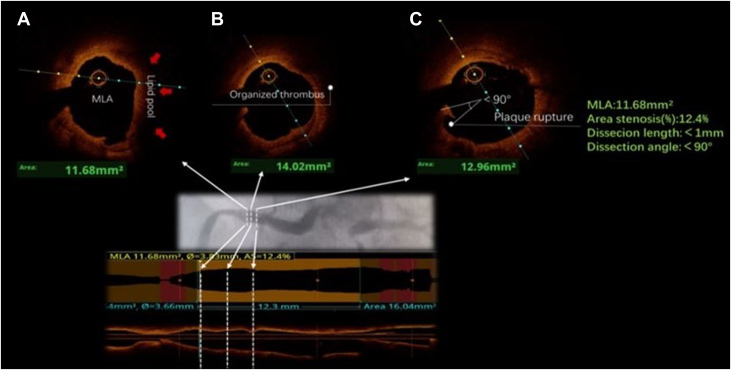
Optical Coherence Tomography Findings of the Proximal Segment of the Right Coronary Artery (A) Cross-sectional image of the right coronary artery with a minimum lumen area (MLA) of 11.68 mm^2^; the red arrows indicate the lipid pool. (B) Cross-sectional image of the organized thrombus, with a lumen area of 14.02 mm^2^. (C) Cross-sectional image of plaque rupture, with a dissection length of <1 mm, dissection angle of <90°, and lumen area of 12.96 mm^2^.

**Figure 3:**
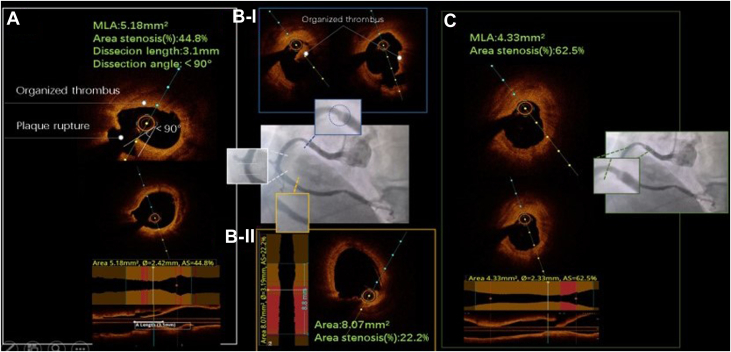
Optical Coherence Tomography Findings in the Middle and Distal Segments of the Right Coronary Artery (A) The organized thrombus and plaque rupture; the minimum lumen area (MLA) is 5.18 mm^2^, the area stenosis rate is 44.8%, the dissection length is 3.1 mm, and the dissection angle is <90°. The organized thrombi (B-I) lumen area: 8.07 mm^2^, area stenosis rate: 22.2% (B-II), MLA: 4.33 mm^2^. (C) Area stenosis rate: 62.5%.

ELCA was employed using a 1.4 mm catheter (40 mJ/mm^2^, 45 Hz) with 4 applications, which significantly reduced the thrombotic burden (Figures 1D to 1F, Video 1, Video 2, Video 3, Video 4, Video 5, Video 6, Video 7, Video 8, Video 9, Video 10). Postprocedural balloon angioplasty restored TIMI flow grade 3. To avoid the necessity for DAPT, stent implantation was deliberately omitted. On postoperative day 1, the patient's platelet count temporarily declined to 28 × 10^9^/L but soon stabilized at 30 × 10^9^/L, without any hemorrhagic complications. Follow-up angiography at 15 days demonstrated resolved right coronary artery stenosis and near-complete thrombus dissolution (Figure 1G). Optical coherence tomography revealed a healed plaque rupture with organized residual thrombus and no significant thrombotic burden (Figures 2 and 3).

## Follow-Up

The patient remained free of angina on clopidogrel monotherapy during the 3-month follow-up. During this period, no major bleeding events or ischemic complications were observed.

## Discussion

The co-occurrence of AA and AMI presents a rare and complex clinical scenario, with the limited data available primarily derived from case reports. Central to this therapeutic challenge is the paradox posed by the interplay between thrombocytopenia associated with AA and the necessity of antithrombotic therapy for patients with AMI. The dual impairment in platelet function and quantity renders conventional antiplatelet aggregation and anticoagulation therapies contraindicated. This unique hematological condition significantly heightens the complexity of clinical decision-making, particularly in devising a suitable coronary revascularization strategy. Currently, international guidelines lack consensus on the optimal management approach for this high-risk population. Here, we report a novel case of a patient with AA with severe thrombocytopenia (platelet count: 28-32 × 10^9^/L) and AMI complicated by a substantial thrombus burden, who was successfully treated with ELCA combined with a leave-nothing-behind PCI strategy. This innovative approach achieved an effective balance between thrombus resolution and bleeding risk mitigation, providing a potential model for addressing similar clinical challenges in the future.

Currently, most experts advocate a hierarchical, descending-ladder antithrombotic strategy for high-risk patients. For individuals with platelet counts between 50 and 100 × 10^9^/L, DAPT comprising aspirin and clopidogrel can be cautiously considered after a thorough evaluation of the thromboembolism-to-bleeding risk ratio. When platelet counts decline to 30 × 10^9^/L, clopidogrel monotherapy is recommended to minimize bleeding risk. Antiplatelet agents should be avoided when platelet counts fall below 30 × 10^9^/L, and PCI is contraindicated in such cases. Dynamic assessments by multidisciplinary teams, including cardiologists and hematologists, are essential to develop individualized treatment strategies.^1^^,^^2^ The 2023 European Society of Cardiology Guidelines recommend PCI as the preferred method of reperfusion for patients with AMI, with coronary artery bypass grafting reserved for situations where PCI is not feasible or when mechanical complications necessitate surgical intervention.^3^ In our patient, a single-drug antithrombotic regimen with clopidogrel was adopted, and dynamic monitoring revealed stable platelet counts with no observed bleeding events. Subsequently, PCI was successfully performed. Coronary angiography identified extensive organized thrombus formation. Conventional management strategies for high thrombus burden—including thrombus aspiration, glycoprotein IIb/IIIa receptor antagonist administration, and intracoronary thrombolysis—partly alleviated no-reflow/slow-flow phenomena but demonstrated limited efficacy in severe cases. After the exclusion of contraindicated treatments, multimodal interventions failed to restore TIMI flow grade 3.

Ultimately, we implemented ELCA, using the photochemical ablation mechanism of a 308-nm ultraviolet laser. Its mechanisms include the photochemical effect of directly cleaving molecular bonds in fibrin and platelet aggregates, the photothermal effect of vaporizing pathological tissue, and the photomechanical effect of generating shockwaves to fragment organized thrombi. These synergistic actions enable efficient removal of organized and high-burden thrombi. Clinical data demonstrate that ELCA exhibits superior feasibility and safety in STEMI treatment,^4^ especially in specific types of lesions,^5^ significantly outperforming the limited efficacy of thrombus aspiration for dense thrombus clearance and the delayed reperfusion/bleeding risks of thrombolytics. ELCA achieves immediate thrombus removal without systemic thrombolysis-related complications. Prospective multicenter registry studies^6^ confirm that under standardized protocols, ELCA significantly reduces vascular dissection perforation rates compared with conventional thrombus aspiration, without increasing the 30-day risk of major adverse cardiovascular events. In a previous study,^7^ ELCA provided significant advantages in treating acute coronary syndrome (ACS) with TIMI flow grade 0, including shortened reperfusion time, improved myocardial perfusion, reduced myocardial injury, and a 2.9% reduction in in-hospital mortality compared with conventional interventions, thereby offering an optimized revascularization strategy for high–thrombus burden lesions. In our patient, ELCA achieved significant thrombus resolution despite the presence of organized thrombus with high thrombus burden, further validating its efficacy in complex thrombotic scenarios. Recent reports^8^ have demonstrated ELCA's efficacy in left main coronary artery lesions without hematologic comorbidities. Our study extends its application to thrombocytopenic AMI, specifically through its implementation in a patient with AMI with concurrent AA and severe thrombocytopenia (platelet count: 28-32 × 10^9^/L). By combining ELCA with a stent-free strategy, we successfully resolved the clinical paradox of balancing antithrombotic efficacy and bleeding risks. This approach not only expands ELCA's indications for high bleeding risk cohorts but also provides personalized therapeutic options for ACS patients with hematologic comorbidities. TIMI flow grade 3 was restored without stent implantation, consistent with evidence-based approaches supporting leave-nothing-behind management in selected ACS cases,^9^ sustained coronary flow itself represents the most potent thrombolytic agent. Follow-up angiography and optical coherence tomography conducted 15 days postprocedure demonstrated significant thrombus resolution, confirming the therapeutic efficacy of ELCA.

Current guidelines do not routinely recommend ELCA for AMI management (in which primary PCI remains the standard therapy^10^). However, expert consensus documents endorse its application in AMI cases with high thrombus burden.^11^ In our patient, conventional thrombus management strategies (including intracoronary thrombolysis, local administration of glycoprotein IIb/IIIa inhibitors, balloon dilation, and thrombus aspiration) were contraindicated or ineffective, with persistent thrombus prompting the ultimate implementation of ELCA for thrombus debulking. ELCA proved highly effective in lysing organized thrombi, while the leave-nothing-behind PCI approach paired with single antiplatelet therapy demonstrated safety in patients at high risk of bleeding. These findings underscore the importance of advancing thrombus-specific interventions and refining individualized antithrombotic protocols for this clinically vulnerable population.

## Conclusions

The combination of ELCA with a stent-free PCI strategy effectively resolved high thrombotic burden and restored coronary flow in a patient with AMI complicated by severe thrombocytopenia, avoiding the need for DAPT and minimizing bleeding risks. This case provides a potential therapeutic paradigm for managing AMI in thrombocytopenic patients.

## Funding Support and Author Disclosures

The authors have reported that they have no relationships relevant to the contents of this paper to disclose.

## Appendix

Video 1 Right Coronary Angiography in the Right Anterior Oblique With Cranial (RAO+CRA) Projection

Video 2 Right Coronary Angiography in the Right Anterior Oblique With Caudal (RAO+CAU) Projection

Video 3 Coronary Angiography in the Caudal (CAU) Projection

Video 4 Coronary Angiography in the Left Anterior Oblique (LAO) Projection

Video 5 Coronary Angiography Demonstrating Thrombus at the Ostium of the Right Coronary Artery

Video 6 After Excimer Laser Coronary Angioplasty Treatment

Video 7 Final Angiographic Results and Restored TIMI Flow Grade 3 in the Right Coronary Artery

Video 8 Follow-Up Right Coronary Angiography at 15 Days Postprocedure

Video 9 Optical Coherence Tomography Assessment of the Proximal Right Coronary Artery at Baseline

Video 10 Optical Coherence Tomography Assessment of the Mid and Distal Right Coronary Artery at Baseline
